# Prostate Cancer Heterogeneous High-Metastatic Multi-Organ-Colonizing Chemo-Resistant Variants Selected by Serial Metastatic Passage in Nude Mice Are Highly Enriched for Multinucleate Giant Cells

**DOI:** 10.1371/journal.pone.0140721

**Published:** 2015-11-04

**Authors:** Lei Zhang, Chengyu Wu, Robert M. Hoffman

**Affiliations:** 1 AntiCancer Inc., San Diego, CA, United States of America; 2 Department of Traditional Chinese Medicine, Nanjing University of Traditional Chinese Medicine, Nanjing, PR China; 3 Department of Surgery, University of California San Diego, San Diego, CA, United States of America; Southern Illinois University School of Medicine, UNITED STATES

## Abstract

In order to further understand the role of tumor heterogeneity in metastasis and chemo-resistance, high metastatic PC-3 human prostate cancer variants were selected by injecting parental PC-3 cells, expressing green fluorescent protein (GFP) in the footpad of nude mice, which then metastasize to inguinal lymph nodes. The PC-3-GFP cells which metastasized to the inguinal lymph nodes were collected and were re-injected to the footpad. After 6 such cycles, the PC-3-GFP cells collected from inguinal lymph nodes (PC-3-GFP-LN) were again injected to the footpad. PC-3-GFP-LN showed 100% metastasis to major lymph nodes (popliteal, inguinal, axillary, and cervical), and 100% metastasis to bone and lung. The percent of giant cell variants was enriched in PC-3-GFP-LN-6 compared to parental cells and increased with each cycle of selection, which in turn had increased metastasis. PC-3-GFP-LN-6 cells were resistant to 5-fluorouracil, doxorubicin and cisplatinum, compared to parental PC-3. However, PC-3-GFP-LN-6 was sensitive to the traditional Chinese medicine (TCM) herbal mixture LQ, similar to the parental cells. These results suggest that PC-3 tumors are heterogenous and that subpopulations of highly metastatic, drug-resistant cells can be step-wise selected using a mouse model of tumor progression.

## Introduction

Cancer cells within a single tumor are heterogeneous, especially with regard to metastasis, chemosensitivity and resistance. Tumor heterogeneity is, therefore, a major problem for effective therapy [1].

Clones from malignant melanoma cells varied greatly in their ability to produce experimental lung metastasis in mice demonstrating heterogeneity of metastatic potential within the population [2, 3].

In a recent study from our laboratory, time-lapse cell-cycle imaging demonstrated that both doxorubicin (DOX) and cisplatinum (CDDP) could induce cell cycle arrest in S/G_2_/M in most of the HeLa cells. However, a subpopulation of the cells could escape the block and undergo mitosis. The subpopulation which went through mitosis subsequently underwent apoptosis, while the cells arrested in S/G_2_/M survived. These results demonstrated heterogeneity of response to cytotoxic drugs [4].

The PC-3 human prostate cancer cell line has been shown to be a promising model to study tumor heterogeneity. The differential morphology of parent PC-3-GFP, PC-3-GFP-circulating tumor cells (CTC) and disseminated PC-3-GFP cells. Disseminated tumor cells (DTC) from multiple metastatic sites were compared. The cultured CTC and DTC from various organs had distinctive morphologies. The distinct morphologies were maintained during in vitro culture [5].

In the present report, we describe isolation of high metastatic variants of PC-3-GFP human prostate cancer cells by serial cycles of isolating lymph node metastasis in nude mouse models. There was a marked shift in the heterogeneous population of cancer cells within this high metastatic variants compared to parental cells that caused drug sensitivity as well as very high frequencies of metastasis.

## Materials and Methods

### Ethics statement

All animal studies were conducted with an AntiCancer Institutional Animal Care and Use Committee (IACUC)-protocol specifically approved for this study and in accordance with the principals and procedures outlined in the National Institute of Health Guide for the Care and Use of Animals under Assurance Number A3873-1. In order to minimize any suffering of the animals the use of anesthesia and analgesics were used for all surgical experiments. Animals were anesthetized with a 20 mL mixture of Ketamine (22–44 mg/kg), Acepromazine (0.75 mg/ kg), and Xylazine (2–5 mg/kg) by intramuscular injection 10 minutes before surgery. The response of animals during surgery was monitored to ensure adequate depth of anesthesia. Ibuprofen (7.5 mg/kg orally in drinking water every 24 hours for 7 days post-surgery) was used in order to provide analgesia postoperatively in the surgically-treated animals. The animals were observed on a daily basis and humanely sacrificed by CO_2_ inhalation when they met the following humane endpoint criteria: prostration, skin lesions, significant body weight loss, difficulty in breathing, epistaxis, rotational motion and body temperature drop. The use of animals was necessary to understand the role of tumor heterogeneity in metastasis and drug resistance. Animals were housed with no more than 5 per cage. Animals were housed in a barrier facility on a high efficiency particulate air (HEPA)-filtered rack under standard conditions of 12-hour light/dark cycles. The animals were fed an autoclaved laboratory rodent diet (S1 ARRIVE Checklist).

### Mice

Athymic nude mice (nu/nu) (AntiCancer Inc., San Diego, CA), 6–8 weeks old, were used in this study. Mice were kept in a barrier facility under HEPA filtration (as noted above). Mice were fed with an autoclaved laboratory rodent diet. All mouse surgical procedures and imaging were performed with the animals anesthetized by subcutaneous injection of the ketamine mixture described above. Isolation of inguinal lymph nodes and the sacrifice of mice was based on tumor size. All animal studies were conducted with an AntiCancer Institutional Animal Care and Use Committee (IACUC)-protocol specifically approved for this study and in accordance with the principals and procedures outlined in the National Institute of Health Guide for the Care and Use of Animals under Assurance Number A3873-1.

### Cells

The PC-3 human prostate cancer cell line expressing GFP used in this study has been described previously [6]. PC-3 cells were grown to 70–80% confluence in RPMI-1640 supplemented with 10% fetal bovine serum and gentamycin (Life Technologies, Inc., Carlsbad, CA, USA), as described previously [6–12].

### GFP gene transduction of PC-3 cells

For GFP gene transduction, 20% confluent PC-3 cells were incubated with a 1:1 mixture of GFPJ-retroviral supernatants of PT67 cells and Ham’s F-12 K (Life Technologies, Inc.) containing 10% fetal bovine serum (Gemini Bio-products) for 72 h. Fresh medium was replenished at this time. PC-3 cells were harvested by trypsin-EDTA 72 h post-transduction and subcultured at a ratio of 1:15 into selective medium that contained 200 μg/ml G418. The level of G418 was increased to 1000 μg/ml stepwise. The brightest PC-3 clones expressing GFP (PC-3-GFP) were selected, combined, and then amplified and transferred by conventional culture methods [6, 13–15].

### Isolation of high metastatic variants of PC-3-GFP

Highly metastatic variants of the PC-3-GFP cell line were selected by injecting parental PC-3-GFP in the footpad of nude mice, which then metastasized to inguinal lymph nodes, from which the cancer cells were collected and cultured in vitro. Those cells were re-injected to the footpad for a total of 6 cycles. The cells from each cycle were named PC-3-GFP-LN-1, PC-3-GFP-LN-2, PC-3-GFP-LN-3, PC-3-GFP-LN-4, PC-3-GFP-LN-5 and PC-3-GFP-LN-6, respectively.

### In vitro drug sensitivity

PC-3-GFP and PC-3-GFP-LN-6 were seeded in 96-well plates. 3-(4,5-dimethylthiazol-2-yl)-2,5-diphenyltetrazolium bromide (MTT) analysis was carried out at 24, 48, and 72 h. Absorbance was read by the Tecan Sunrise Microplate Reader using the Magellan software (Tecan Systems). Data were processed using Microsoft Excel [11]. CDDP (Pharmaceutical Buyers Intl., New York, NY) was treated at 1 μM/ml, 2.5 μM/ml, 5 μM/ml and 10 μM/ml. DOX (Pharmaceutical Buyers Intl.) was treated at 10 μM/ml, 20 μM/ml, 25 μM/ml and 50 μM/ml. 5-fluorouracil (5-FU) (Pharmaceutical Buyers Intl.) was treated at 50 μM/ml, 100 μM/ml, 200 μM/ml and 500 μM/ml. TCM-LQ (16, 17) was treated at 0.072 mg/ml, 0.18 mg/ml, 0.36 mg/ml and 0.9 mg/ml. CDDP, DOX and 5-FU were dissolved in PBS. PBS was as the control. With regard to preparation of TCM LQ, we refer to our previous publication which was explained in detail (16, 17). Cells were exposed to the drugs for 24, 48 and 72 hours in 96-well plates.

### Statistical analysis

The Student’s *t-*test was performed to generate *p*-values comparing PC-3-GFP and PC-3-GFP-LN-6 growth curves for drug treated and untreated control cells. A *p*-value of <0.05 was considered significant [11].

## Results and Discussion

**Fig 1:**
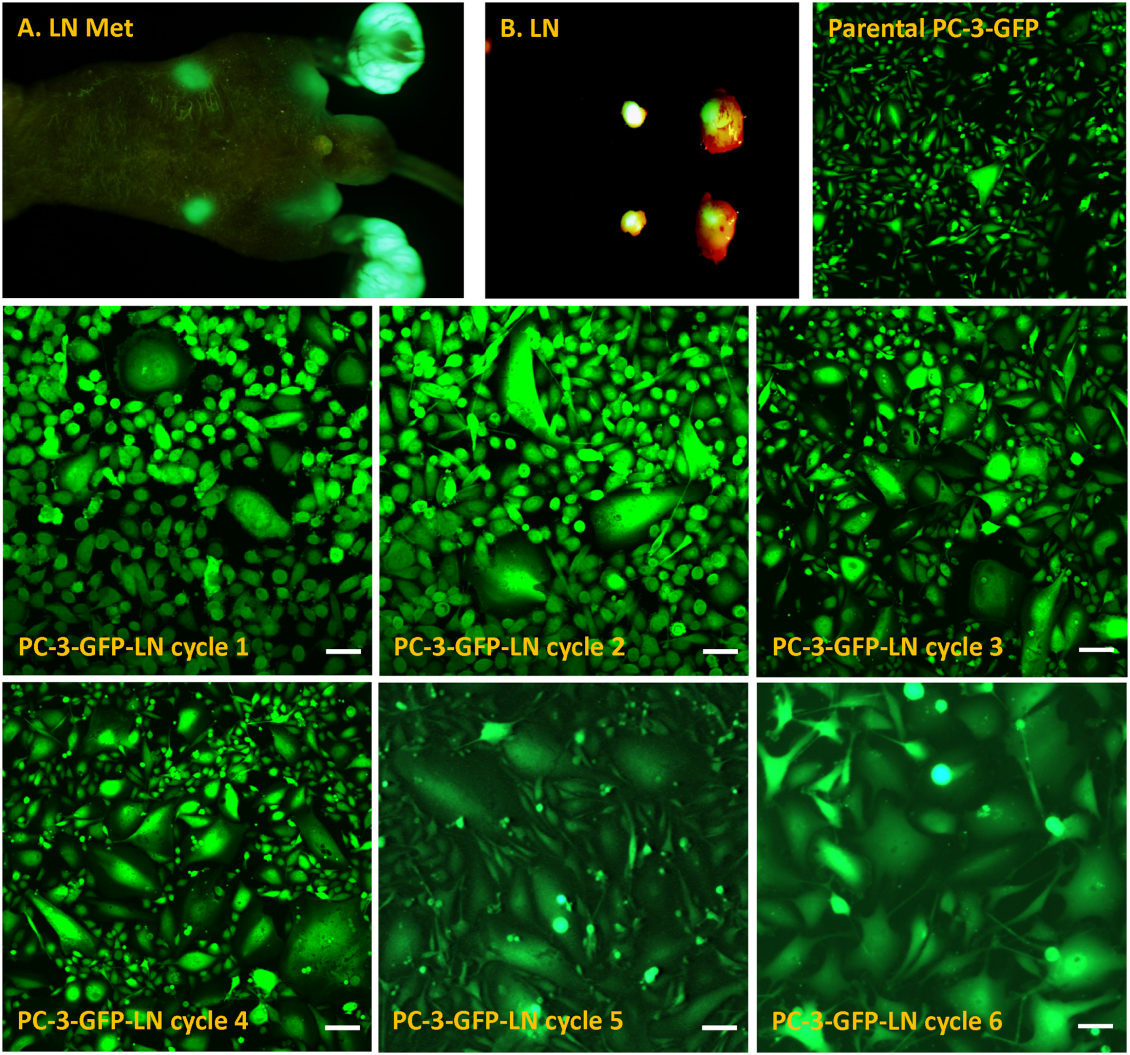
PC-3-GFP cells were injected to the footpad of mice. After 3 weeks, PC-3-GFP developed spontaneous metastasis in the popliteal lymph node and inguinal lymph node. The metastatic PC-3-GFP cells of the inguinal lymph node were collected and re-injected in the footpad to develop variants with increased metastatic potential. The cells in the inguinal node were collected and re-injected the footpad. After 6 such cycles of re-injection and selection of metastasis, the selected cell line developed 100% of metastasis in the lung, bone, inguinal node, axillary node, and cervical node (A&B). The morphology of in vitro cultured metastatic PC-3-GFP-LN cells, from each cycle, cultured from inguinal lymph node metastasis and parental PC-3-GFP are shown. The giant cell number was enriched with the cycle number, indicating that giant cells are more aggressive and highly metastatic cells.

PC-3-GFP cells were injected to the footpad of nude mice. After 3 weeks, PC-3-GFP developed spontaneous metastasis in the popliteal and inguinal lymph nodes. The metastatic PC-3-GFP cells from the inguinal lymph node were collected and re-injected in the footpad to develop further metastasis. The metastatic potential of PC-3-GFP increased with each cycle (Table 1). After 6 cycles of the above-procedure, the selected cell line developed 100% metastasis in the lung, bone, inguinal node, axillary node, and cervical node (Table 1). The morphology of in vitro cultured metastatic PC-3-GFP-LN cells from inguinal lymph node metastasis (Cycle 1–6) and parental PC-3-GFP was compared. The number of giant cells was enriched with each selection cycle indicating that giant cells have increased metastatic potential (Fig 1).

**Table 1:** Metastatic frequency. Metastasis frequency after in vivo metastasis selection cycles one to six of PC-3-GFP. After 6 cycles of selection, the PC-3-GFP-LN6 variant subline developed metastasis in the lung, bone, inguinal node, axillary node, and cervical node in all mice.

	1	2	3	4	5	6
**Popliteal LN**	**30%**	**100%**	**100%**	**100%**	**100%**	**100%**
**Inguinal LN**	**10%**	**40%**	**100%**	**100%**	**100%**	**100%**
**Bone**	**0**	**0**	**10%**	**100%**	**100%**	**100%**
**Lung**	**0**	**0**	**0%**	**10%**	**60%**	**100%**
**Axillary LN**	**0**	**0**	**0**	**10%**	**60%**	**100%**
**Cervical LN**	**0**	**0**	**0**	**10%**	**50%**	**100%**

**Fig 2:**
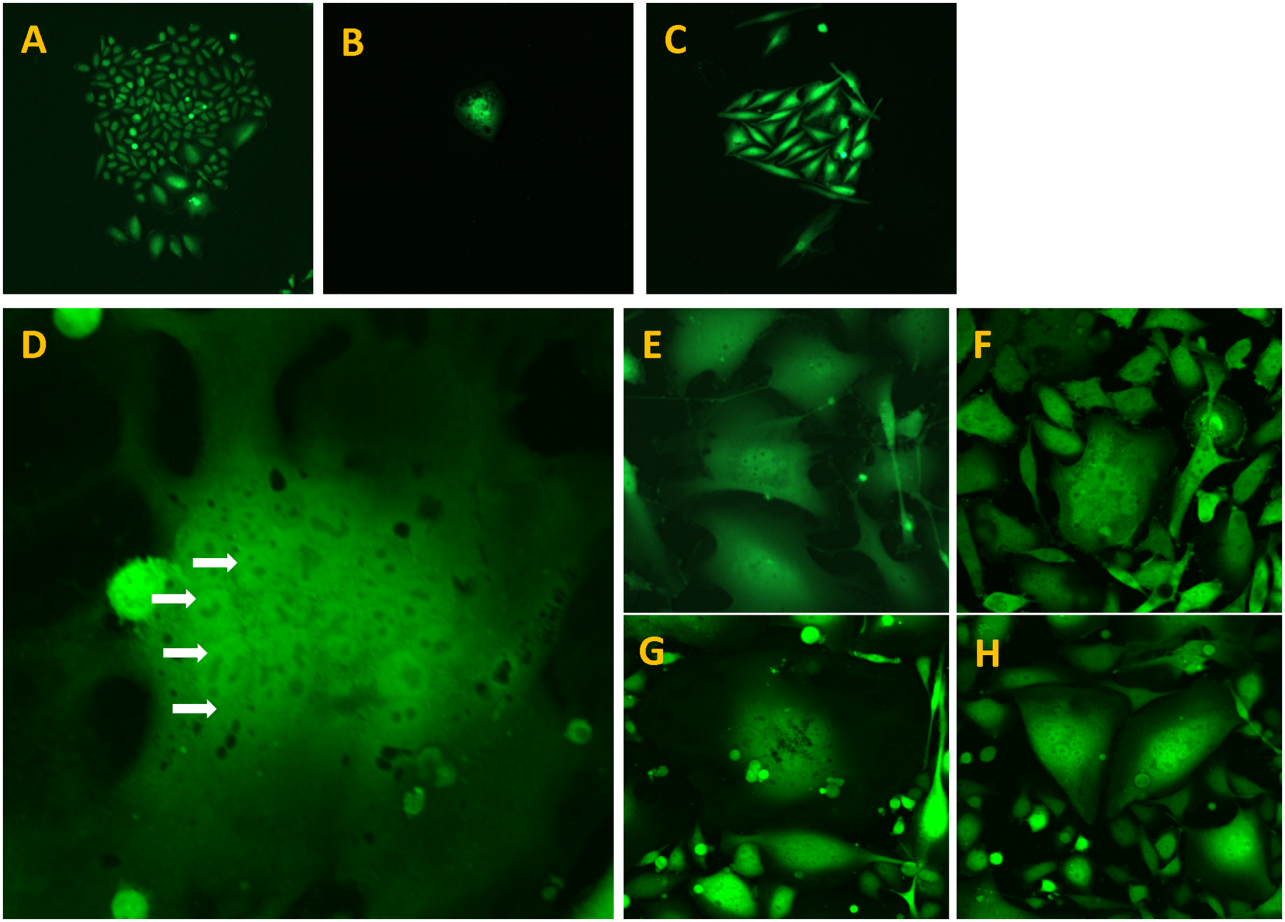
Cellular morphology of PC-3-GFP-LN-6 (A-C). PC-3-GFP-LN-6 contained round, spindle and giant cells. The giant cells contain multiple nuclei (D-H). The number of nuclei per cell ranges between 2–22. Arrows show some of the multiple nuclei in a giant cell.

The PC-3-GFP-LN-6 variant contained round, spindle and giant cells (Fig 2). The giant cells contain multiple-nuclei. The number of nuclei per cell ranges between 2–22 (Fig 2). The giant cells may contain more information and higher ability of metastasis than single-nuclei cells. Giant cell number increased with each selection, which in turn became more metastatic, presumably due to the increased number of giant cells.

**Fig 3:**
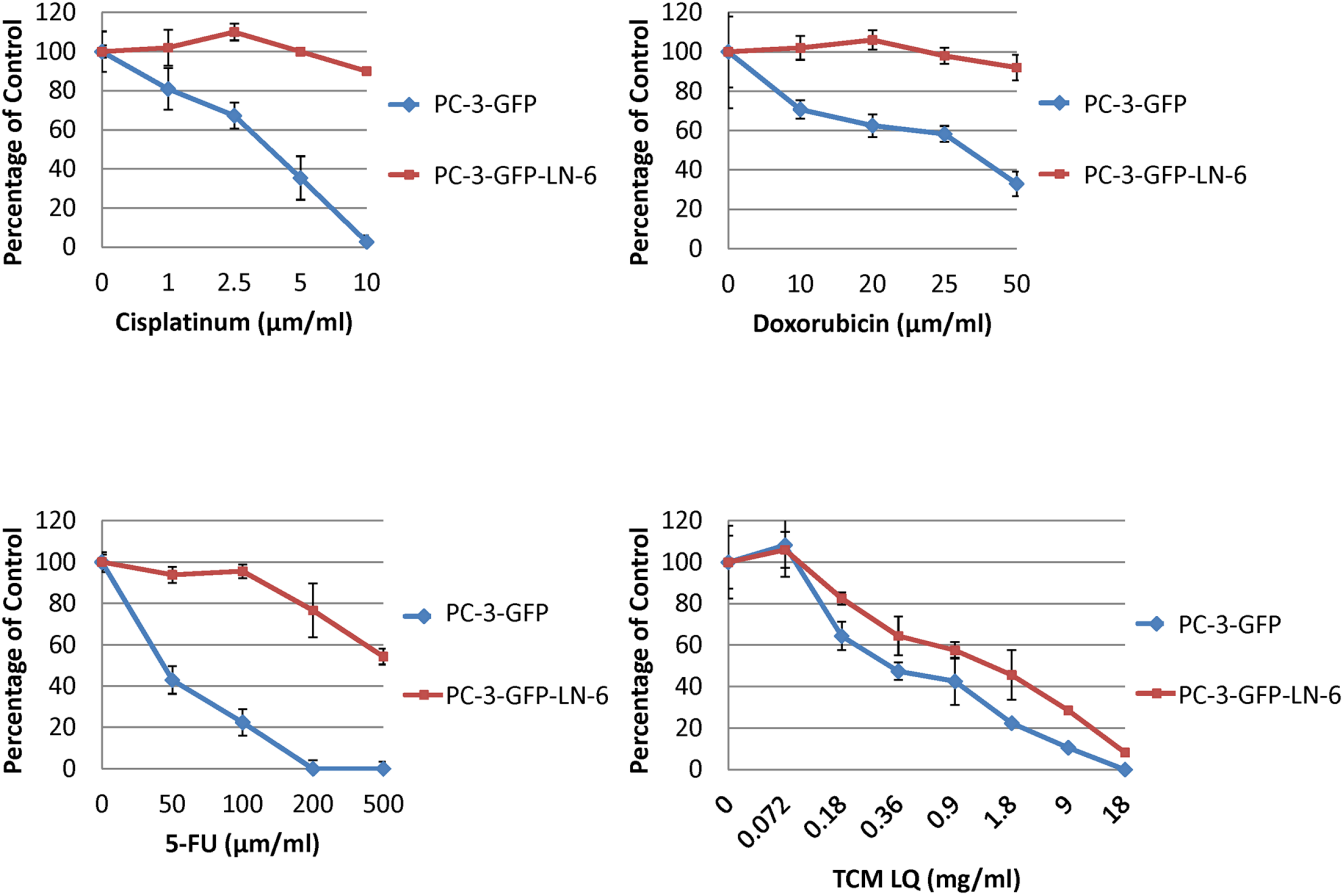
Sensitivity of the PC-3-GFP-LN3-6 cells to 5-fluorouracil (5-FU), cisplatinum (CDDP), doxorubicin (DOX), and traditional Chinese medicine herbal mixture LQ. PC-3-GFP-LN-6 was highly-resistant to all chemotherapeutic drugs tested compared to the parental PC-3-GFP cells (*p*<0.01). PC-3-GFP-LN-6 only showed similar sensitivity to the parental cell line PC-3-GFP when treated with TCM LQ.

Sensitivity of the PC-3-GFP-LN6 cells and parental cells to 5-fluorouracil (5-FU), CDDP, DOX and the traditional Chinese medicine (TCM) herbal mixture LQ [15–18] was tested. PC-3-GFP-LN3-6 variants were highly-resistant to all chemotherapeutic drugs tested compared to the parental cells (*p*<0.01). PC-3-GFP-LN-6 only showed similar sensitivity to the parental cell line to TCM LQ (Fig 3).

Metastasis frequency to various organs was determined after each cycle of selection (Table 1). After 6 cycles of re-injection, the PC-3-GFP-LN variant subline developed metastasis in the lung, bone, inguinal node, axillary node, and cervical node after footpad injection (Table 1). Metastasis to popliteal and inguinal lymph nodes increased to 100% by passage 2 and 3, respectively. Bone metastasis increased to 100% by passage four and lung metastasis increased to 100% by passage 6. There were no axillary or cervical lymph node metastases until passage 4 which did not reach 100% until passage 6 (Table 1). Thus the ability of PC-3-GFP to metastasize to some metastatic sites required more in vivo selection than metastasis to other sites.

The results of the present study indicate the high degree of heterogeneity of PC-3-GFP tumors and the importance of heterogeneity to metastatic capability to various sites. It is also noteworthy that the high metastatic variants were highly drug resistant. Metastasis and drug resistance of PC-3-GFP circulating tumor cells studies were studied previously [19]. Although PC-3-GFP CTC were more metastatic than parental PC-3-GFP, the CTC did not become drug resistant [11] as did the PC-3-GFP-LN-6 high metastatic variant in the present report. These results raise questions as to whether CTC give rise to metastasis.

The multiple-nuclei giant cells were enriched with each selection cycle and became predominant in the selected highly-metastatic PC-3-GFP-LN6, drug-resistant cell line and therefore may play an important role in metastasis and drug resistance. Targeting those cells with TCM “LQ” suggests a new approach to the treatment of metastatic prostate cancer, since the highly metastatic drug resistant PC-3-GFP-LN-6 was sensitive to LQ.

Thus, metastatic selection shifted the heterogeneous population of the PC-3 cells to be highly enriched in giant cells. It is noteworthy that variants metastatic to bone were selected. It is also noteworthy that selection for high metastasis also resulted in high resistance to 3 very different types of chemotherapy drugs. The present studies demonstrate the dangerous consequences of selective pressure on tumors for metastasis and drug resistance.

### Dedication

This paper is dedicated to the memory of Dr. A.R. Moossa.

## Supporting Information

S1 ARRIVE Checklist ARRIVE checklist. (PDF)
